# The modernization of cardiology in Iraq (From apprenticeship to competency-based approach)

**DOI:** 10.1016/j.amsu.2022.103669

**Published:** 2022-04-27

**Authors:** Ameen M. Mohammad

**Affiliations:** Department of Medicine, College of Medicine, University of Duhok, Kurdistan region, Iraq

## Abstract

• This report is unprecedent in treating a shift in medical training, particularly cardiology, in Kurdistan and whole Iraq.
• It attempts to motivate a scientific debate to advance post graduate learning process in cardiology.
• It distinguishes the apprenticeship from the competency-based approach and encourages a move towards the latter.
• It links this shift with the development of competencies determining cardiological advancement.

Educational systems produce different learning outcomes with vital importance for the practice of the acquired knowledge. Traditional systems place attention on time during which learners acquire knowledge in scientific fields. Advanced ones are directed towards the development of competencies on the part of learners. The core distinction between the two systems regards the outcome of the learning process; while learners necessarily develop competencies in advanced systems, in traditional ones, they do not necessarily do so. This has undenied implications for the practice of knowledge acquired by learners in the two systems [1].

The world witnesses a promising movement from traditional towards advancing educational systems. The movement is understandably neither easy nor global. But it is happening in various fields of knowledge. Modern societies are better positioned in terms of capacities and willingness to undertake changes in educational systems [1,2]. The movement towards an advanced system primarily focusing on the development of competencies in scientific fields is driven by modern societies. In Middle Eastern countries, this movement has not been materialized as traditional systems are still in place widely. Iraq constitutes only an instance in this regard. Although it will not be easy, an attempt to move away from traditional educational systems in Iraq is nonetheless worthwhile. This article is aimed at providing some insights in this context, focusing particularly on the cardiological aspect of medical education.

In medicine, traditional systems produce learners who likely do not acquire knowledge necessary for an effective practice of the field. The learning process completes mainly with reference to a planned duration. Learners participate in a collective learning process that is basically driven by lecturers [3]. Their individual and community needs for learning are not sufficiently taken into consideration. Whether they develop needed competencies pale in comparison with the duration of the learning process. With a formal completion of the learning, learners may be misled about their proficiencies and have only a limited willingness to keep learning. The implication of this approach for the practice of medicine is obvious: practitioners are not competent in navigating the complexities of human body and its nature. The traditional systems produce some competent practitioners also mainly due to their individual efforts though [3,4].

The Iraqi and Kurdistan Region Boards of Medical Specialities were founded in 1988 and 2009 respectively to act as formal institutions to accredit training programs in various medical fields. Then after, the Cardiology Training Program (Board) was launched within these two institutions. The different aspects of the program were developed by national professionals. These include the development of curriculum and assessment procedures that are based on the apprenticeship-traditional approach. Such approach has many vulnerabilities in Iraq and Kurdistan like the deficiency in the quality of graduates and outcomes, marginalization of different patients' needs, dominancy of doctor-centered concept and lack of many competencies domains and documentations. Therefore, the program needs to come under several essential modification to move away from this approach. Some of these are concerned with the curriculum and its delivery, the assessment mechanisms of postgraduate students, and the admission procedure of candidates. In Iraq, medical learning should increasingly move towards the development of competencies in various fields. Cardiology should begin to aim at mastering proficiencies adopted by the American Board of Medical Specialities in line with the competency-based approach. They are the clinical skills, medical knowledge, professionalism, practice-based learning, system-based practice, and interpersonal communication [5].

A competency-based approach to learning is expectedly related with the development of certain attributes in the practitioners of cardiology. A competent cardiologist has an independent style of learning. This means that the development of proficiency in the field does not necessarily rest on the educational system. It rather becomes a continued process of self-learning through classical schools, wards and beyond. The source of learning is not limited to the material given in compulsory classes and places but extends to various scientific platforms and outcomes outside the formal education and after its completion [3].

Such a competent cardiologist should have a curiosity of researcher. The world of science changes rapidly from different perspectives. There are always challenges to old theories and confirmations of new ones. Competent cardiologists engage in research to test their knowledge, acquire new insights, and provide contributions to scientific communities. They become aware of new developments in their fields and serve patients in effective ways consequently. Given the rapidity of advancement in science, a cardiologist disengaged from research is vulnerable to follow outdated wisdom in the treatment of novel circumstances. This is destined to bring heavy costs for subjects of treatment, something that is at the core of medicine to avoid [6].

Competency is also about developing skills to successfully guide patients, teach learners, and train enthusiasts. Patients can have significant knowledge limitations about their circumstances and need to make risky decisions on some occasions. A competent cardiologist guides them into making the most appropriate ones. The role of guidance is not confined to patients. Teaching learners in an advanced manner is another part of the guiding role that a competent cardiologist has. This same role applies in the context of trainings provided for practicing fans of cardiology. In the absence of competency by a guiding cardiologist, the knowledge presented to patients, learners, and trainees is not appropriately useful [7].

Heart teamwork is an essential dimension of cardiological competency. It naturally provides pathways to the enrichment of knowledge, the consolidation of capacities, and the effective accomplishment of missions. A competent cardiologist is convinced about the benefits associated with heart team work and plays strongly in multiplying them. Such a cardiologist becomes a member of teams at various possible levels, benefits from the experiences of counterparts in cardiovascular specialities, and contributes to the enrichment of others' knowledge, particularly in grey zone of cardiovascular medicine and hybrid procedures [8].

Cardiological competency extends to the skill of decision-making. The practice of cardiology involves making decisions that have vital implications on subjects. Making the right decision according to unique circumstances of subjects constitutes a fundamental proficiency in cardiology. It takes into consideration a holistic examination of circumstances and broad evidence-based knowledge. A competent cardiologist has a recognized reputation of having the needed proficiency to make right decisions. Making such appropriate scientifically driven decisions is of unique significance, particularly given the safety of patients and some wider implications for communities [9].

While technology maintains positively contributing to the advancement of medicine from various sides, the recent introduction of Cath lab and other technological instruments into cardiology is associated with practical risks and specific qualifications. They necessitate from cardiologists advanced capacities to succeed and continuously scrutinize their operation. The cardiovascular operations and interventions, by itself are of high risks nature. A competent cardiologist is well prepared to successfully intervenes and maintains the quality inside the laboratory. This covers competencies about the complete familiarization of instruments, the accurate monitoring of operations, the right reaction to emerging uncertainties. Finally, it extends to the application of the best available medical knowledge into practice ([10,11]).

The development of these competencies in the practice of cardiology establishes a promising prospect for the future of the field in Iraq. The contemporary learning system of cardiology in the country employs traditional approaches that undermine the advancement of the field. Given all the benefits linked with advanced educational systems, it is necessary to increasingly move away from traditional ones. This article attempted to provide a combination of competencies that cardiologists should develop according to advanced systems. It suggests that these competencies prepare the ground for the modernization of cardiology and recommends a shift of the focus towards their development in Iraq.

## Ethical approval

N/A.

## Source of funding

No funding to declare.

## Author contribution

AMM: designed and draft the study, and conducting the version of the manuscript.

## Trail registry number

Name of the registry:
Unique Identifying number or registration ID:
Hyperlink to your specific registration (must be publicly accessible and will be checked):

## Guarantor

Ameen M Mohammad.

## Declaration of competing interest

None.

## Provenance and peer-review

Not commissioned, externally reviewed.

## Declaration of competing interest

No conflicts of interests.
